# Nanoparticle-Based Delivery Systems for Synergistic Therapy in Lung Cancers

**DOI:** 10.3390/bioengineering12090968

**Published:** 2025-09-09

**Authors:** Zicheng Deng, Ali Al Siraj, Isabella Lowry, Ellen Ruan, Rohan Patel, Wen Gao, Tanya V. Kalin, Vladimir V. Kalinichenko

**Affiliations:** 1 Phoenix Children’s Research Institute, Department of Child Health, University of Arizona College of Medicine—Phoenix, Phoenix, AZ 85004, USA; 2 School of Life Sciences, College of Liberal Arts & Sciences, Arizona State University, Tempe, AZ 85281, USA; 3 Arizona College of Osteopathic Medicine, Midwestern University, Glendale, AZ 85308, USA; 4 Phoenix Children’s Center for Cancer and Blood Disorders, Phoenix Children’s Hospital, Phoenix, AZ 85016, USA; 5 Division of Hematology and Oncology, Department of Child Health, University of Arizona College of Medicine-Phoenix, Phoenix, AZ 85004, USA; 6 Division of Neonatology, Phoenix Children’s Hospital, Phoenix, AZ 85016, USA

**Keywords:** lung cancer, nanoparticle, drug resistance, drug delivery, gene therapy, synergistic therapy

## Abstract

Lung cancer remains the leading cause of cancer-related mortality worldwide, with conventional treatments often limited by systemic toxicity, different tumor sensitivity to the drugs, and the emergence of multidrug resistance. To address these challenges, nanoparticle-based delivery systems have emerged as an innovative strategy, enabling the simultaneous transport of multiple agents, including chemotherapeutic drugs and expression vectors, to enhance treatment efficacy and overcome tumor resistance. This review explores various nanocarrier platforms, such as liposomes, solid lipid nanoparticles, polymeric micelles, and inorganic nanoparticles, specifically designed for lung cancer therapy. Synergistic effects and physicochemical properties of therapeutic agents must be carefully considered in the design of nanoparticle-based co-delivery systems for lung cancer therapy. We highlight the applications of these nanoparticle systems in drug–drug, gene–gene, and drug–gene co-delivery approaches. By addressing the limitations of traditional therapies, nanoparticle-based systems offer a promising avenue to improve outcomes in patients with lung cancers.

## 1. Introduction

Lung cancer is the leading cause of cancer-related death, with an estimated 2.2 million new cases and 1.8 million deaths globally in 2020 [1]. Tobacco exposure is considered the main risk factor for lung cancer, but environmental exposures and genetics also play significant roles in lung cancer incidence and mortality [2]. Chemotherapy remains the main treatment for lung cancer and is often the only option for some lung cancer subtypes [3,4,5]. However, most of the chemotherapy agents indiscriminately kill rapidly growing cells, both tumor and normal cells, leading to severe side effects that significantly affect patients’ quality of life [6]. Moreover, the extensive damage to the normal cells makes chemotherapy unsuitable for patients with severely compromised organ systems [7]. Another major challenge in cancer chemotherapy is the development of drug resistance, which is responsible for over 90% of cancer-related deaths [8]. Cancer cells can acquire genetic alterations during treatment, leading to resistance to chemotherapeutic agents [9]. For example, lung cancer tumors with Epidermal Growth Factor Receptor (*EGFR)* mutations often acquire drug resistance [10]. In addition to mutations, other mechanisms contributing to drug resistance include increased drug efflux, enhanced DNA repair capabilities, production of growth factors, derivation of oncogenic transcription factors, and elevated xenobiotic metabolism [8,11,12,13]. Therefore, there is a critical need for new therapeutic strategies to reduce damage caused by chemotherapy to normal cells and to overcome drug resistance.

Nanotechnology is widely used in various fields, including energy generation [14], environmental protection [15], gene therapy [16,17], and precision medicine [18,19]. In cancer therapy, nanotechnology represents an innovative approach that enhances therapeutic efficacy while minimizing the adverse effects of anticancer drugs [20,21,22]. In physiological environments, hydrophobic drugs tend to aggregate, which reduces their solubility, bioavailability, and therapeutic efficacy [23]. Likewise, nucleic acids are extremely unstable and can be readily degraded by enzymes such as RNases and DNases in biological fluids, limiting their clinical application [24,25]. Nanoparticle delivery systems overcome these challenges by encapsulating hydrophobic drugs or condensing nucleic acids within protective carriers. These carriers prevent aggregation, shield therapeutic agents from enzymatic degradation, and prolong systemic circulation, thereby improving physicochemical stability, biopharmaceutical properties, and overall therapeutic outcomes. Various types of nanoparticles (NPs) can be engineered with different sizes, charges, structures, and surface functionalization to achieve effective therapeutic delivery to cancer cells. For instance, numerous studies have shown that NPs within the size range of 20–150 nm tend to accumulate more effectively at tumor sites [26]. This tumor selectivity is due to the enhanced permeability and retention (EPR) effect, a unique pathophysiological phenomenon of the solid tumor microvasculature that promotes nanoparticle accumulation. Because tumor blood vessels are often irregular, loosely connected, and highly permeable, nanoparticles in the range of roughly 20–150 nm can more readily exit the circulation and enter tumor tissue compared to normal vasculature. In addition, tumors typically lack efficient lymphatic drainage, which means that once nanoparticles penetrate the tumor site, they tend to remain there for extended periods rather than being rapidly cleared [27,28]. Therefore, carefully controlling the size of NPs within this optimal range is critical in NP designs for cancer therapy. In addition to size, surface charge plays a key role in enhancing the efficiency of nanoparticle-mediated drug delivery to cancer cells. Due to the “Warburg Effect,” tumor cells produce large amounts of lactate through aerobic glycolysis, and the cross-membrane movement of the lactate leads to the removal of labile inorganic cations, resulting in a net negative surface charge on cancer cells [29,30]. In contrast, normal cells typically maintain a neutral or slightly positive surface charge as they rely on mitochondrial oxidative phosphorylation to generate adenosine triphosphate (ATP) [31,32]. Consequently, NPs with positively charged surfaces have demonstrated high targeting efficiency to cancer cells through electrostatic interactions [30,31]. Beyond passive targeting based on physicochemical properties of NPs, such as size and charge, the large surface-to-volume ratio of NPs allows for the functionalization of specific ligands, enabling active targeting of cancer cells. For example, the folate receptor is one of the most widely known receptors overexpressed in cancer cells. NPs functionalized with folic acid can be delivered to the cancer cells more efficiently due to the folic acid/folate receptor interaction [33,34]. Moreover, NPs have unlocked the potential for delivering therapeutic nucleic acids, both DNA and RNA, to specific cells [35,36,37,38]. In gene therapy, nanocarriers are essential for protecting nucleic acids from degradation, overcoming biological barriers, and crossing cell membranes to target tumor cells [39,40].

**Figure 1:**
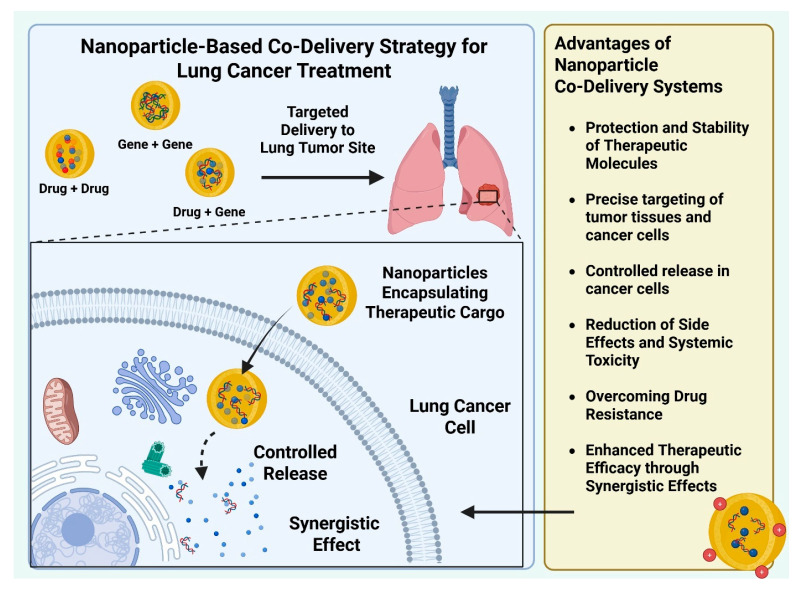
Different co-delivery combinations for lung cancer treatment and their advantages. Created with BioRender.com.

NPs are emerging as a promising tool for synergistic lung cancer therapy [41]. Recent reviews on nanoparticle-based therapies for lung cancer have primarily focused on specific nanoparticle types or individual therapeutic strategies [7,42,43,44]. In contrast, this review specifically focuses on nanoparticle-based co-delivery strategies for lung cancer synergistic therapy. To ensure relevance, we included studies that investigated the simultaneous delivery of multiple therapeutic agents and explored the molecular mechanisms underlying their synergistic effects, particularly how these strategies can overcome the limitations of conventional single-agent drug or gene delivery. We placed emphasis on studies that provided detailed experimental or preclinical evidence of enhanced therapeutic efficacy achieved through co-delivery. This methodological approach enabled us to highlight the rationale, design principles, and therapeutic advantages of nanoparticle-based co-delivery systems in the context of lung cancer synergistic therapy. Synergistic therapy involves combining multiple therapeutics within a single delivery system to achieve three key goals: (1) enhance treatment efficacy, (2) reduce systemic toxicity, and (3) prevent drug resistance (Figure 1). Previous studies demonstrated that combination therapy is more effective than using a single therapeutic and can also help delay the onset of acquired drug resistance in lung tumor cells [45]. In lung cancer therapy, inhalation administration offers significant advantages by increasing local drug concentration in the lungs while minimizing accumulation of the drugs in non-target organs. For example, a recent study reported the development of inhalable NPs simultaneously loaded with erlotinib (ERL) and curcumin, demonstrating a promising therapeutic approach for the direct delivery of these agents to treat non-small cell lung cancer (NSCLC) [46]. Typically, the synergistic therapy using NPs can be categorized into drug–drug, drug–gene, and gene–gene combinations, depending on the types of cargo encapsulated into the NPs. For lung cancer therapies, it is crucial to design NPs that can efficiently encapsulate and deliver multiple cargos while also understanding the synergistic effects between multiple therapeutics. In this review, we provide complementary perspectives on co-delivery strategies in two sections. Section 2 highlights various nanoparticle platforms and their advantages for drug and gene delivery, serving as a guide for selecting suitable carriers. Section 3 focuses on the therapeutic rationale for combining multiple agents within a single system, with particular emphasis on synergistic effects and the underlying molecular mechanisms.

## 2. Different Types of Nanoparticles for Simultaneous Delivery of Therapeutic Agents in Lung Cancers

**Figure 2:**
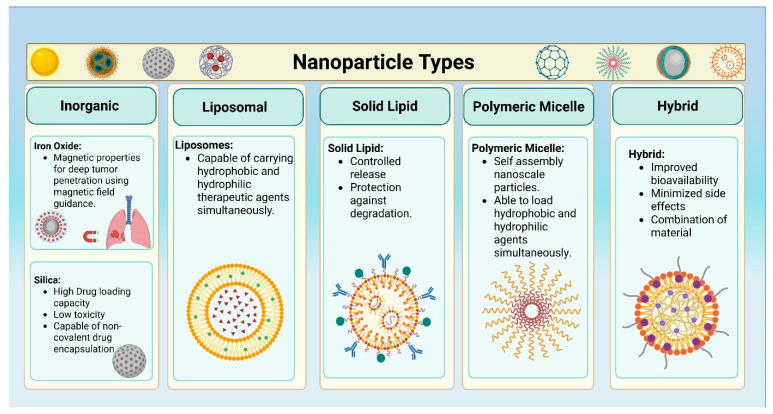
Different types of nanoparticles capable of simultaneously delivering drugs and nucleic acids for lung cancer therapy. Created with BioRender.com.

Co-delivery systems utilizing NPs represent an innovative approach in the treatment of lung cancers, offering a promising strategy for enhancing therapeutic efficacy [47]. Currently, various types of NPs have been developed to effectively and simultaneously encapsulate multiple therapeutic agents, including both chemotherapeutic drugs and nucleic acids, enabling synergistic cancer therapy (Figure 2). Beyond encapsulation, researchers also engineered the NPs to control the spatial and temporal distribution of the cargos for controlled release and stronger synergistic effects [48]. The choice of NP type typically depends on the nature of the cargo. Cationic materials are widely used for gene delivery due to their ability to condense nucleic acids into NPs through electrostatic interactions. NPs containing amphiphilic molecules are commonly employed in the delivery of hydrophobic drugs due to their ability to stabilize hydrophobic cargoes [49,50]. Overall, designing an appropriate NP co-delivery system requires selecting the NP type to match the specific therapeutic cargo.

### 2.1. Liposomes

Liposome-based drug delivery systems have emerged as a highly effective approach for enhancing therapeutic efficacy in various applications, including the treatment of lung cancer. Liposomes are spherical hollow vesicles containing single or multiple phospholipid bilayers with an aqueous center [51]. The phospholipid molecule contains both a hydrophobic tail and a hydrophilic head, enabling liposomes to spontaneously assemble into NPs when dispersed in water, forming spherical bilayers that resemble the lipid bilayer of cellular membranes [52]. A key advantage of liposomes is the dual-compartment architecture consisting of the hydrophilic aqueous core and hydrophobic bilayer that simultaneously encapsulate hydrophilic and hydrophobic therapeutic agents, facilitating synergistic delivery [53]. Liposomes also possess tunable physicochemical properties that are critical for their performance. Their surface charge can range from highly negative (−60 mV) to highly positive (+43 mV), largely determined by lipid composition, with cationic lipids such as stearylamine (SA) conferring positive charge and anionic lipids like dicetyl phosphate (DCP) generating negative charge [54]. Particle size typically ranges from 20 to 200 nm, an optimal range that enhances tumor accumulation and extravasation via the EPR effect [55]. Furthermore, liposome stability is maximized under low-temperature conditions and mildly acidic environments (~pH 6.5), helping to maintain structural integrity [56]. By loading different drugs or bioactive molecules within a single vesicle, these NP systems can target multiple cellular pathways, reduce off-target effects, and improve therapeutic outcomes. Recently, Ma et al. engineered chondroitin sulfate-modified liposomes to co-deliver berberine (BBR, high hydrophilicity) and magnolol (MAG, low hydrophilicity), demonstrating enhanced antitumor activity in lung cancer models through synergistic pro-apoptotic effects [57]. In addition, this nanoparticle system exhibited sustained drug release compared to free drugs, with cumulative release rates for BBR and MAG reaching 73% and 42% at 24 h, and 93.53% and 69.29% at 84 h, respectively [57]. Furthermore, cationic molecules can be incorporated into liposomes to confer a positive surface charge and enable electrostatic condensation of nucleic acids (e.g., siRNA, mRNA, minicircle DNA) for simultaneous delivery of drugs and nucleic acids [58,59]. Zhang et al. leveraged this strategy by encapsulating survivin-specific small interfering RNA (siRNA) (complexed with protamine) alongside paclitaxel (PTX) in liposomal NPs. A synergistic inhibitory effect on cancer cell growth was demonstrated by enhanced cytotoxicity and increased intracellular uptake. This NP system achieved potent tumor suppression in lung cancer cells at reduced PTX doses, highlighting the potential of this combinatorial therapy [60]. Beyond cargo versatility, liposomes offer other important advantages, such as enhanced drug stability, controlled release kinetics, protection of nucleic acids from degradation, and functionalization of NP surfaces for active targeting [61].

Typically, the liposome preparation involves the following main stages: (1) dissolving lipids in organic solvents; (2) drying lipids from the organic solvents; (3) hydrating the lipids in an aqueous medium; (4) downsizing the liposome product; and (5) purification and analysis [62,63]. Common methodologies for liposome preparation with cargo include the thin-film hydration method, the ethanol injection method, the reverse phase evaporation method, and the detergent depletion method [64]. For example, Qu et al. prepared cationic liposomes using the thin-film hydration technique with subsequent co-delivery of anticancer drug Docetaxel (DTX) and BCL-2 siRNA for lung cancer therapy [65]. In this study, which used an A549 lung cell-bearing xenograft tumor model, the DTX/siRNA NP treatment exhibited a sustained release and remarkable tumor regression with a 100% survival rate [65]. In another study focusing on NSCLC treatment, the reverse-phase evaporation method was used to co-load vinorelbine and cisplatin into liposomes, yielding enhanced antitumor activity and reduced systemic toxicity [66]. Despite the promise, liposomal systems face limitations, including high production costs, poor stability during extended storage, short shelf life, and scalability hurdles [63,67]. Despite these challenges, liposome-based co-delivery NP platforms remain a cornerstone of personalized lung cancer therapy, with ongoing research focused on optimizing multifunctional designs for clinical translation.

### 2.2. Solid Lipid Nanoparticles

Solid lipid nanoparticles (SLNs) represent a promising platform for advanced drug co-delivery systems, with growing potential in cancer therapy. Their high biocompatibility enables effective navigation of physiological barriers, while their lipid matrix enhances stability, protects sensitive therapeutics from degradation, and enables controlled release profiles compared to conventional carriers [68]. The amphiphilic nature of SLNs allows simultaneous incorporation of hydrophilic and lipophilic drugs, broadening their therapeutic versatility. Furthermore, SLNs can be engineered for targeted delivery, minimizing systemic toxicity and improving site-specific efficacy [69,70]. The surface charge of SLNs is also tunable and can be primarily adjusted by the choice of lipid type (e.g., stearic acid, Precirol) and surfactant selection. For instance, Tween 80 generally produces higher zeta potential values compared to Poloxamer 188 [71]. Particle size can likewise be controlled by varying the concentrations of lipids and surfactants. In addition, the stability of SLNs is strongly influenced by the crystallinity of the lipid matrix and potential polymorphic transitions, which determine their ability to retain encapsulated drugs over time. SLNs employ diverse techniques to encapsulate therapeutic agents. In high-pressure homogenization, drugs are mixed with molten lipid, emulsified in an aqueous surfactant solution, and cooled to form submicron-sized NPs [70]. Alternatively, the solvent evaporation method was used to dissolve lipids in organic solvents, emulsifying the mixture, and evaporating the solvent under reduced pressure. While this method avoids thermal stress, residual solvents may introduce variability in particle size [72]. Both encapsulation strategies enhance drug solubility and bioavailability by efficiently loading hydrophilic and lipophilic agents. One key advantage of SLNs is the ability to release drugs at a controlled rate. SLNs modulate drug release through matrix crystallinity, degradation, diffusion, and environmental responsiveness. The solid core acts as a sustained-release reservoir, enabling gradual drug diffusion for prolonged therapeutic effects [73,74]. In a recent study, SLNs co-loaded with PTX and curcumin demonstrated sustained release kinetics in lung cancer models, reducing toxicity while maintaining efficacy [75]. Additionally, pH sensitivity and enzymatic degradation can be exploited to tailor SLNs for tumor-specific microenvironments [76,77]. Gupta et al. developed pH-responsive SLNs for the simultaneous delivery of PTX and ERL that exhibited significantly enhanced drug release under acidic conditions [78]. Accelerated growth of lung tumor cells and their altered metabolism, especially the tendency to rely on glycolysis even when oxygen is sufficient (the Warburg effect), results in an accumulation of lactic acid. Consequently, the extracellular space in lung tumors becomes more acidic, often around pH 6.5 to 6.9. This acidic environment can be strategically exploited in the design of a nanoparticle system that releases therapeutic agents specifically in response to low pH, improving tumor-targeted efficacy [78]. SLNs enable the combinatorial delivery of drugs and the nucleic acid cargo. Han et al. engineered transferrin (Tf)-coated SLNs to co-deliver plasmid DNA with doxorubicin (DXR), achieving targeted lung cancer cell uptake and synergistic therapeutic effects [79]. Surface modifications, such as polyethylene glycol (PEG) conjugation, further enhance stability and reduce off-target effects [73]. Li et al. combined PEG and Tf functionalization to encapsulate both DTX and baicalin, yielding sustained release, superior tumor suppression, and minimized systemic toxicity in lung cancer models [80].

### 2.3. Polymeric Micelles

Polymeric micelles have emerged as a versatile platform for co-delivery systems in cancer therapy, leveraging their unique amphiphilic structure to enhance therapeutic efficacy. These micelles are self-assembled nanoscale particles composed of block polymers, featuring a hydrophobic core (for encapsulating poorly soluble drugs) and a hydrophilic shell (for colloidal stability and stealth properties) [81]. This dual-compartment architecture enables simultaneous loading of hydrophilic and hydrophobic agents, making them ideal for combinatorial therapy. Recently, Xu et al. developed polymeric NPs formed by methoxy poly(ethylene glycol)–poly(ethylenimine)–poly(l-glutamate) (mPEG-OEI-PLG) polymers for pulmonary delivery of DXR and cisplatin (CP) in the same NP cargo [82]. This noninvasive approach reduced systemic toxicity while improving therapeutic outcomes in metastatic lung cancer formed by the injection of B16F10 cells into C57BL/6 male mice [82]. DOX and DCPP exhibited release rates of 72% and 78% at pH 5 over 72 and 120 h, respectively, and the drugs remained in lung tissues for as long as 7 days [82]. Polymeric micelles can be tailored into diverse architectures, including reverse micelles (hydrophilic core, lipophilic shell) and mixed micelles (blends of surfactants or lipids). Reverse micelles are widely used to solubilize hydrophilic drugs in organic media, study membrane proteins, and serve as nanoreactors for enzymatic reactions [83,84,85]. Conversely, mixed micelles, composed of multiple polymers, offer enhanced thermodynamic stability, higher drug-loading capacity, and dual-drug delivery capabilities. For example, the mixed micelle NP delivery system based on D-alpha-tocopherol polyethylene glycol 1000 succinate (TPGS) (PEG2000-DSPE and vitamin-E TPGS) was capable of delivering PTX and parthenolide, achieving potent chemosensitization and antitumor efficacy in NSCLC models [82,86]. In comparison with other nanocarriers, the benefits of polymeric micelles include easier preparation, smaller size, and higher drug loading capacity. Polymeric micelles are made from nontoxic biodegradable polymers like PEG, poly (lactic-co-glycolic acid) (PLGA), polyvinyl imine (PEI), polycaprolactone (PCL), polyvinyl alcohol (PVA), Poly (β-Amino ester) (PBAE) [87,88,89,90]. These polymers allow for safer metabolism and elimination in the body. Polymeric micelles generally self-assemble when placed in aqueous environments; thus, their production can be easily scaled up [91]. In comparison to liposomes or inorganic NPs, polymeric micelles have increased stability and decreased immune clearance [92]. Polymeric micelles also exhibit highly adaptable surface charge, which can be precisely tuned by the type of polymer used [93]. Incorporation of cationic polymers (e.g., PBAE) imparts a positive charge, enhancing cellular uptake and facilitating the delivery of nucleic acids or anticancer drugs. Conversely, neutral polymers such as PEG help balance or reduce surface charge, thereby improving colloidal stability, minimizing nonspecific interactions, and extending systemic circulation [93]. In addition, the overall size of polymeric micelles is strongly influenced by the hydrophobicity of the core-forming block, which governs self-assembly and drug encapsulation efficiency [88]. Polymeric micelles can also be designed for stimuli-responsive drug release, which allows for a more controlled drug distribution in targeted tissues. For example, the release of NP contents can be controlled via pH, redox conditions (e.g., glutathione-responsive release), enzyme-sensitive methods (for example, based on the activity of metalloproteinases or hyaluronidase), temperature, electric fields, magnetic fields, and light [94]. Recently, pH-sensitive micelles were capable of co-delivering PTX and siRNA specific to survivin [95]. The PEG shell detached in the acidic tumor microenvironment, with a release rate reaching 86% after 72 h, thereby enhancing cellular uptake and endosomal escape for synergistic NSCLC therapy [95].

### 2.4. Inorganic Nanoparticles

Inorganic NPs are widely utilized in co-delivery systems due to their versatility as platforms for diverse therapeutic applications [96]. For example, iron oxide NPs are an ideal material for targeted delivery due to their magnetic properties and tunable size [97]. Iron oxide NPs can be synthesized through various approaches, including hydrothermal synthesis, thermal decomposition, and coprecipitation, each yielding distinct physicochemical properties [98]. Hydrothermal and thermal decomposition methods provide fine control over particle size and crystalline phase, producing uniform nanoparticles with narrow distributions, typically between 4 and 100 nm. By contrast, coprecipitation is favored for its simplicity and scalability but often results in nanoparticles with higher surface energy and limited stability, making them prone to aggregation. To address this drawback, surface modification strategies are commonly employed, such as coating IONPs with stabilizing polymers (e.g., PEG, PVP), surfactants, or small organic molecules, which enhance colloidal stability, reduce aggregation, and improve size dispersity [99]. When scaled down to the nanoscale, iron oxide NPs exhibit superparamagnetism, retaining no magnetization in the absence of an external magnetic field. This property enables precise localization and deep tissue penetration of the NPs via magnetic field guidance, enhancing tumor-targeted delivery [100]. Additionally, superparamagnetic iron oxide nanoparticles (SPIONs) can absorb near-infrared (NIR) light to generate localized heat, enabling hyperthermia therapy for cancer treatment [30,31]. Inorganic NPs can be chemically modified to improve interactions with drugs or nucleic acids, boosting delivery efficiency through covalent or non-covalent conjugation strategies [101,102]. Ngema et al. developed conjugated linoleic acid (CLA)-coated SPIONs for PTX delivery in lung cancer therapy [103]. CLA, a small molecule compound with anticancer activity, synergized with hydrophobic anticancer drug PTX to enhance tumor suppression, demonstrating the potential of iron oxide-based inorganic NPs as a co-delivery system for cancer therapy [103]. This NP delivery system showed a maximum cumulative release of 94% within 12 h under acidic conditions, accompanied by pronounced anti-proliferative effects on cancer cells [103]. Similarly, silica NPs, which constitute another class of inorganic NP carriers, offer high drug-loading capacity, thermal stability, low toxicity, and non-covalent drug encapsulation capabilities [96]. Zhang et al. engineered mesoporous silica nanoparticles (MSNs) with high surface area and pore volume to co-deliver CP and oleanolic acid. CP directly induced cancer cell apoptosis in an A549-derived lung cancer model, while oleanolic acid sensitized tumor cells to overcome CP resistance [104], supporting the utility of silica-based co-delivery systems for lung cancer therapy.

### 2.5. Hybrid Nanoparticles

Hybrid NPs are gaining attention as powerful tools in cancer therapy due to their ability to combine multiple materials, leveraging the unique advantages of each component to enhance delivery efficiency, improve bioavailability, and minimize side effects. Since they are composed of two or more distinct types of nanomaterials, they exhibit an exceptionally broad range of physicochemical characteristics [105]. By integrating complementary material components, these systems achieve enhanced tunability in parameters such as size, surface charge, stability, and drug-loading capacity. The physicochemical traits of hybrid nanoparticles can be precisely tailored through controlled selection and ratio adjustment of each constituent material, enabling the design of customized combinations that optimize therapeutic performance, improve biodistribution, and enhance functional versatility for specific biomedical applications [105]. For example, iron oxide NPs can be coated with lipids to co-deliver antigens and adjuvants for synergistic lung cancer immunotherapy. In a recent study, lipid-coated iron oxide NPs (IONP-C/O@LP) were developed as a nanovaccine to co-encapsulate peptide antigens and cytosine–phosphate–guanine (CpG) DNA adjuvants, significantly inhibiting tumor growth and improving survival in mouse models derived from TC-1 tumor cells, which originated from lung epithelial cells [106]. Lipid–polymer hybrid NPs are also explored for lung cancer therapy in animal models. Wu et al. engineered aptamer-decorated lipid–polymer hybrid NPs to co-deliver DTX and CP. The aptamer functionalization enhanced lung cancer cell targeting, demonstrating the potential of combination therapy to achieve synergistic effects, reduce toxicity, and overcome drug resistance [107]. Similarly, pH-sensitive lipid–polymer hybrid NPs were designed using adipic acid dihydrazide (ADH) as a pH-responsive linker and hyaluronic acid (HA) for tumor targeting, exhibiting accelerated drug release under acidic conditions (pH 6.0). These NPs co-deliver ERL and bevacizumab (BEV), offering a promising strategy for NSCLC therapy [108]. Recent advances include biomimetic hybrid NPs that integrate biological materials. For instance, protein-lipid core–shell NPs co-delivering genistein (GNS) and all-trans retinoic acid (ATRA) were developed as inhalable formulations, exhibiting an initial rapid release (55% within 8 h) followed by a slower, sustained release reaching 65% at 72 h. These particles reduced lung tumor size and biomarkers, highlighting their potential for localized therapy [109]. Zhang et al. further combined cancer cell membranes, lipoic acid-modified polypeptide micelles, and charge-reversal liposomes to deliver DTX together with PGAM1 siRNA (siPGAM1), resulting in a burst release of both therapeutic agents. This multifunctional NP system synergistically inhibited tumors in xenograft models by regulating glycolysis and prolonging animal survival without notable toxicity [110].

## 3. Lung Cancer Synergistic Therapy Using Nanoparticle-Based Co-Delivery Systems

**Figure 3:**
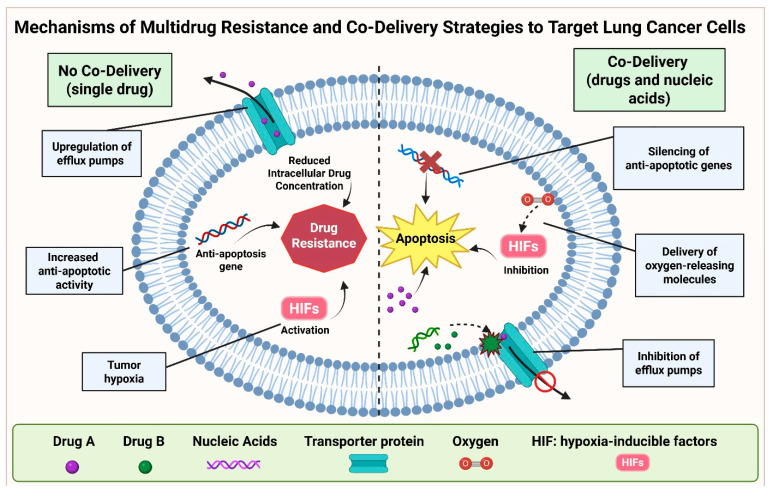
The mechanisms of multidrug resistance in lung cancer cells and the nanoparticle co-delivery strategies used to overcome the drug resistance. Created with BioRender.com.

Single therapeutic treatments are often inefficient in treating lung cancers. Innovative NP approaches can overcome this limitation by exploring the synergistic effects of two or more therapeutic agents that target different mechanisms of tumor cell survival or resistance (Figure 3). These strategies show considerable promise in overcoming multidrug resistance (MDR) in lung cancer cells, as they interfere with multiple resistance pathways simultaneously, thereby enhancing therapeutic efficacy [111]. Common mechanisms of drug resistance include the overexpression of efflux transporters, defective apoptotic pathways, and hypoxic microenvironments [6]. Among these mechanisms, drug efflux pumps are a major contributor to chemotherapy resistance. P-glycoprotein (P-gp), for example, is overexpressed in cancer cells and actively transports chemotherapeutic agents out of tumor cells, thereby reducing intracellular drug concentrations and therapeutic effectiveness [112,113]. Suppressing these efflux pumps represents a promising strategy to improve treatment outcomes. Recently, the general control non-repressed 5 (GCN5) transcript was reported to downregulate P-gp expression, weakening the efflux activity in drug-resistant cancer cells. NP delivering DXR and GCN5 siRNA has been shown to markedly inhibit tumor growth in MDR breast cancer models [114]. In addition, cancer cells often evade apoptosis by upregulating anti-apoptotic proteins such as Bcl-2 and other inhibitors of apoptosis proteins (IAPs) [113]. To counteract this, researchers have co-delivered survivin-specific small hairpin RNAs (shRNA) with chemotherapeutic agent PTX to overcome drug resistance in lung cancer cells [115]. They reported that delivering survivin shRNA can downregulate survivin gene expression (a member of the IAP family), significantly improving the therapy outcomes [115]. Hypoxic environments within tumors activate hypoxia-inducible factors (HIFs), promoting drug resistance in cancer cells [116]. HIF-1α enhances the expression of ATP-binding cassette (ABC) transporters, which directly efflux chemotherapeutics out of cancer cells. HIF-1α also upregulates IAPs, helps to maintain cancer stem cells, and regulates autophagy, thereby enhancing cell viability and resistance in lung tumors [117,118,119]. To reverse hypoxia-induced drug resistance, Li et al. developed liposomal NPs co-delivering the anticancer drug ERL and perfluorooctyl bromide, a perfluorocarbon capable of dissolving large amounts of oxygen. This combination effectively reversed hypoxia-driven drug resistance in lung cancer cells [120].

### 3.1. Nanoparticle-Based “Drug–Drug” Co-Delivery Systems

The hydrophobicity of many therapeutic agents is a significant challenge in cancer chemotherapy, limiting their solubility in biological buffers and reducing bioavailability. NPs with hydrophilic outer shells offer a promising solution by protecting hydrophobic drugs from aggregation, prolonging the half-life of unstable compounds, and enabling targeted delivery and controlled release at tumor sites [121]. These NP systems typically require a hydrophobic inner core to stabilize poorly water-soluble drugs and a hydrophilic surface to ensure biocompatibility and prolonged circulation. For example, gefitinib, a first-generation EGFR tyrosine kinase inhibitor, is widely used in chemotherapy for various cancers. Similarly, quercetin, a flavonoid with significant antitumor activity, also exhibits poor water solubility. The hydrophobic nature of both compounds restricts their clinical applications [122]. Amphiphilic poly(lactic-co-glycolic acid)–polyethylene glycol (PLGA–PEG) polymer NPs can encapsulate both of these hydrophobic drugs, enabling synergistic treatment of lung cancer in a PC-9 lung cancer cell-derived model. PLGA–PEG NPs co-encapsulating these drugs were found to have an average diameter of about 120 nm and a negative surface charge, aiding in NP stability. Encapsulation efficiencies exceeded 80% for both drugs, and in vitro release studies demonstrated sustained release over 4–6 h, correlating with enhanced cytotoxicity against PC-9 lung cancer cells [122]. Similarly, sorafenib and crizotinib, both FDA-approved anticancer drugs, suffer from low bioavailability due to their hydrophobic nature. Encapsulation of these drugs in PEG-PCL-PEG amphiphilic polymer nanoparticles, with an average size of 30 nm and a negative surface potential, achieved an encapsulation efficiency greater than 90%. This co-delivery system significantly reduced tumor metabolism while minimizing toxicity to normal organs [123].

Conversely, hydrophilic chemotherapeutic agents, despite their aqueous solubility, often exhibit non-specific biodistribution, leading to systemic toxicity and reduced therapeutic efficacy. Co-delivery of these agents with complementary hydrophobic drugs can mitigate these challenges. For example, CP is a platinum-based chemotherapeutic agent that is widely used in lung cancer treatment. Encapsulation of CP with hydrophobic drugs using layer-by-layer lipid NPs offers a promising new approach [124]. Fan et al. utilized this NP design to encapsulate CP together with oridonin, where the hydrophobic oridonin was incorporated into the PLGA core, while CP was encapsulated in an outer lecithin layer. The encapsulation of oridonin and CP using this method resulted in a particle size of about 150 nm with a negative surface charge. This co-delivery system resulted in enhanced in vitro cell toxicity and in vivo antitumor effects compared to single drug-loaded NPs [124]. Another strategy to overcome solubility mismatches between co-delivered drugs involves modifying hydrophilic drugs to enhance their hydrophobicity [125]. Recently, CP has been conjugated with fatty acids to form a hydrophobic CP prodrug, which was later encapsulated with hydrophobic agents such as etoposide and PTX into PLGA-PEG polymeric NPs [125,126,127]. This formulation exhibited high encapsulation efficiency and maintained storage stability at 4 °C for at least two weeks. Co-delivery did not substantially interfere with either the loading capacity of the drug or alter the physicochemical properties of the carrier, supporting the feasibility of achieving synergistic drug ratios for therapeutic effect [125,126,127]. This strategy is also applicable for the co-delivery of CP with another hydrophilic drug. For instance, Yang et al. developed a hyaluronic acid–cisplatin/polystyrene–polymetformin dual prodrug for precise NP delivery of CP and metformin into lung cancers. The micelle-like structure of the dual-prodrug system promoted self-assembly of the NPs with a particle size of about 160 nm and zeta potential of −17.4 mV and showed synergistic antitumor effects in lung cancer models [128].

### 3.2. Nanoparticle-Based “Gene-Gene” Co-Delivery Systems

Gene therapy has emerged as a promising approach for lung cancer treatment due to the genetic basis of tumor development and progression. Understanding the genetic aberrations involved in lung cancer cell genesis has facilitated the targeted delivery of specific genes to inhibit cancer progression and prevent metastasis. Gene therapies typically rely on cationic materials, which carry positive charges to electrostatically bind and condense negatively charged nucleic acids (plasmid DNAs, small interfering RNAs, mRNAs, n-coding RNAs, or microRNAs) into stable NPs. The design requirements for such systems are straightforward: NPs must incorporate cationic agents capable of efficiently encapsulating and protecting genetic cargo while facilitating targeted delivery. For example, loss-of-function mutations in caspase 8 (CASP8) and downregulation of microRNA clusters miR-29A-B1 and miR-34A microRNAs have been identified as critical drivers of apoptosis resistance in NSCLC. A recent study employed chitosan-PLGA hybrid NPs to co-deliver three therapeutic plasmids encoding miR-29A-B1, miR-34A, and CASP8. These nanoparticles have a hydrodynamic diameter of 139 nm and contain positively charged polymers, which are important for nucleic acid condensation and protection. This combinatorial gene delivery system significantly restored apoptotic signaling in tumor cells, overcoming drug resistance and enhancing therapeutic efficacy [129].

### 3.3. Nanoparticle-Based “Drug-Gene” Co-Delivery Systems

Another promising NP approach in cancer therapies is the combination of chemotherapeutic drugs with expression vectors or RNAs. Integrating gene therapy approaches with chemotherapy can enhance cancer treatment by simultaneously targeting multiple oncogenic pathways. Such NP co-delivery systems can inhibit key oncogenic processes like tumor angiogenesis, metastasis, and tumor cell proliferation, offering a promising synergistic approach to enhance therapeutic efficacy [130]. For instance, the co-delivery of Bcl-2 siRNA and DXR using cationic liposome-incorporated poly(ε-caprolactone)/chitosan nanofibers demonstrated a synergistic effect, significantly increasing cancer cell death in vitro utilizing the A2780/AD human cancer cells [131]. Similarly, the co-delivery of DXR and survivin siRNA via polymeric NPs significantly enhanced cytotoxicity against lung cancer cells in vitro using B16F10 cells [132]. In these studies, DXR induced cancer cell death, while siRNA inhibited the expression of anti-apoptotic genes, preventing cancer cells from evading apoptosis. Another example is the co-delivery of TUBB3 siRNA (targeting βIII-tubulin, a protein associated with microtubule formation and cancer resistance) and DTX using redox-responsive NPs composed of PEG-disulfide-PLGA and oligo(β-aminoesters). This formulation produced spherical nanoparticles approximately 150 nm in size with a negative surface charge, exhibiting high encapsulation efficiencies and controlled release behavior. The simultaneous delivery of both agents enhanced apoptosis and reversed drug resistance in lung cancer cells, significantly increasing DTX cytotoxicity in NSCLC models by overcoming resistance mechanisms [133]. In another study, PTX and VEGF siRNA were co-encapsulated into lipid-based NPs with a diameter of approximately 150 nm, leveraging the synergistic effects of PTX’s cytotoxicity and VEGF siRNA’s anti-angiogenic properties. This dual therapy approach efficiently inhibited tumor growth while minimizing systemic toxicity in NCI-H460 cells from lung cancer models [134]. The studies covered in this review are summarized in Table 1.

## 4. Current Challenges and Future Perspectives

Nanoparticle-based co-delivery systems have shown great promise in the development of novel therapies for lung cancer treatment. While several NPs are currently in active clinical trials for lung cancer therapy (NCT05703971, NCT04789486, NCT02769962), these trials primarily involve traditional single-agent NPs rather than co-delivery systems. To date, no co-delivery NP system has progressed to clinical trials, highlighting the considerable challenges that remain before clinical applications in patients with lung cancer. One major challenge is the difficulty in scaling up the production of co-delivery NPs. Compared to traditional NP delivery systems, which already face limitations in yield, co-delivery systems involve additional processing steps, making large-scale production more complex. Furthermore, it remains unclear whether the precise ratio of co-delivered therapeutics can be reliably maintained during industrial-scale manufacturing. Additionally, most studies on co-delivery NPs in lung cancer research focus on short-term therapeutic outcomes in cell cultures or small animal models. While these studies provide valuable insights, they do not fully address long-term toxicity, biodistribution, or therapeutic efficacy in humans. Future research approaches should include more comprehensive preclinical evaluations, such as large-animal studies and long-term toxicity assessments, to better investigate the outcomes. NP anticancer therapy-related future studies should not only focus on designing novel co-delivery NP systems but also consider the feasibility of large-scale production and their long-term biological impact. Researchers should collaborate closely with industry partners to address production challenges and ensure that these NP systems can be translated into clinically viable therapies. Long-term studies on the safety and biocompatibility of co-delivery NP systems are essential to bridge the gap between laboratory research and clinical applications. By addressing these challenges, NP co-delivery systems can move closer to becoming a transformative approach in lung cancer therapies.

## 5. Conclusions

Lung cancer continues to be a leading cause of cancer-related deaths worldwide. Recent advances in nanotechnology have shown great promise in addressing the challenges associated with traditional anticancer treatments, such as systemic toxicity and drug resistance. Nanoparticle-based co-delivery systems, which combine multiple therapeutic agents such as chemotherapeutic drugs and nucleic acids, have demonstrated the potential to enhance treatment efficacy compared to single-agent NPs. This improvement is attributed to the synergistic effects of the co-delivered therapeutics and their ability to overcome drug resistance. Although promising preclinical evidence supports the potential of these innovative NP systems for lung cancer therapies, further research is needed to optimize NP designs, improve the safety and efficacy, and advance the NP co-delivery systems toward clinical translation. Currently, the NP co-delivery systems are still in early stages of development and have not entered clinical trials for lung cancer therapy. Nevertheless, nanoparticle-based co-delivery systems represent a promising approach in the future of precision medicine, offering new opportunities for more effective and targeted lung cancer treatments.

## Figures and Tables

**Table 1:** Nanoparticle-co delivery systems for lung cancer synergistic therapy.

NP	Cargo	Efficacy	Toxicity	Reference
Liposome	Berberine (BBR) and Magnolol (MAG)	An in vitro study showed improved cellular uptake and enhanced cytotoxicity on lung cancer A549 cells.	Reduced systemic toxicity (No significant body weight and pathological changes observed in treated mice)	[57]
Liposome	Survivin-specific siRNA and paclitaxel (PTX)	Enhanced siRNA uptake by approximately 1.4-fold. Increased Cytotoxicity on NCI-H460 lung cancer cells.	A decreased PTX dosage was required to limit toxic effects.	[60]
Liposome	Docetaxel (DTX) and BCL-2 siRNA	Inhibited cell proliferation in A549 and H226 lung cancer cells in a time-dependent and dose-dependent manner, with significant apoptosis observed.	100% survival rate for mice treated with lipo-DTX/siRNA.	[65]
Solid lipid	Paclitaxel (PTX) and Erlotinib (ERL)	Enhanced cellular uptake and improved inhibition of NCI-H23 cells.	Not mentioned.	[78]
Solid lipid	Docetaxel (DTX) and baicalin (BA)	The tumor volumes in mice treated with Tf-D/B-SLNs were significantly smaller compared to free drugs.	Reduced systemic toxicity (No significant body weight change observed in treated mice)	[80]
Polymeric micelles	Doxorubicin (DOX) and cis-platinum (CDDP)	Co-NPs exhibited higher anti-tumor efficiency for metastatic lung cancer than single treatments of DOX or CDDP.	Reduced systemic toxicity (No obvious body weight loss and organ damage observed in treated mice)	[82]
Polymeric micelles	Paclitaxel (PTX) and survivin siRNA	Accumulation of nanoparticles at the tumor site led to pronounced inhibition of tumor growth in treated mice.	Reduced systemic toxicity (Minor effect on normal cell progression)	[95]
Inorganic nanoparticles	Conjugated linoleic acid (CLA) and Paclitaxel (PTX)	Highly suppressed A549 cell proliferation.	Not mentioned.	[103]
Inorganic nanoparticles	Cisplatin and oleanolic acid	Increased Apoptosis of cancer cells in treated mice	Reduced systemic toxicity (No obvious body weight loss observed in treated mice)	[104]
Hybrid nanoparticles	Peptide antigen and adjuvant (CpG DNA)	IONP-C/O@LP treatment synergistically improved the efficacy of DC activation and enhanced antigen-specific T cell responses. Significantly inhibited tumor growth and remarkably prolonged animal survival rates.	Reduced systemic toxicity (No obvious body weight loss and organ damage observed in treated mice)	[106]
Hybrid nanoparticles	Docetaxel prodrug (DTXp2) and cisplatin (DDP)	LPHNs showed significantly higher distribution in the tumor than free drugs and profound tumor inhibition ability.	No systemic toxicity. Hematological parameters, including blood urea nitrogen (BUN), serum aspartate aminotransferase (AST), and alanine aminotransferase (ALT) levels, were all within the normal range for LPHNs groups.	[107]
Hybrid nanoparticles	Erlotinib (ERL) and bevacizumab (BEV)	Strong tumor suppression with markedly smaller tumor volumes.	Low cardio-renal toxicity.	[108]
Hybrid nanoparticles	Genistein (GNS) and all-trans retinoic acid (ATRA).	The treated group exhibited reduced lung lesion numbers, a lower average count of microscopic metastatic lung adenomatous foci, and decreased tumor biomarker levels in vivo.	Reduced systemic toxicity (No significant body weight change observed in treated mice)	[109]
Hybrid nanoparticles	Docetaxel (DTX) and Phosphoglycerate mutase 1 (PGAM1) siRNA (siPGAM1)	NP highly accumulated in A549 tumor xenografts in vivo.	Reduced systemic toxicity (No obvious body weight loss and organ damage observed in treated mice)	[110]
Liposome	Erlotinib (ERL) and perfluorooctyl bromide	Reversal of hypoxia-driven drug resistance in cancer	Not mentioned	[120]
Lipid	Cisplatin (CP) and Oridonin	Increased cell accumulation due to the cell membrane and lipid-based NP. With an increase in stability.	Cell toxicity was studied using A549/DDP cells, and found that blank NP did not showcase any cell toxicity. Loaded NP increases in toxicity as the concentration of the drugs increases.	[124]
Polymeric Micelle	Hyaluronic acid-cisplatin/polystyrene and Polymetformin	Dual-Prodrug exhibited self-assembly with antitumor effects in lung cancer models.	Not mentioned.	[128]
Chitosan and poly(lactic-co-glycolic acid) (PLGA)	miR-29A-B1, miR-34A, and CASP8.	Delivering three agents effective at restoring apoptotic signaling in tumor cells.	The highest amount of dose given at 80 ug/mL induced an 88% survival rate of cells	[129]
cationic liposome-incorporated poly(ε-caprolactone)/chitosan	Bcl-2 siRNA and Doxorubicin (DXR)	A2780/AD was utilized and demonstrated the co-loaded NP enhanced cancer cell death.	Not mentioned.	[131]
PEG-disulfide-PLGA and oligo(β-aminoesters).	TUBB3 siRNA and Docetaxel (DTX)	Enhanced the cytotoxic effect on NSCLC by bypassing the drug resistance mechanism.	Gene knockdown was successful and showcased no observable cytotoxicity to nearby cells.	[133]
Lipid	Paclitaxel (PTX) and VEGF small interfering RNA (siRNA)	Controlled tumor growth using NCI-H460 cells.	A low dose of PTX was required to reduce toxicity levels.	[134]

## Data Availability

Not applicable.

## Abbreviations

The following abbreviations are used in this manuscript:

Adenosine triphosphate	ATP
Enhanced permeability and retention	EPR
Epidermal growth factor receptor	EGFR
Nanoparticles	NPs
Non-small cell lung cancer	NSCLC
Solid lipid nanoparticles	SLN
Berberine	BBR
Magnolol	MAG
Small interfering RNA	siRNA
Paclitaxel	PTX
Docetaxel	DTX
Polyethylene glycol	PEG
Poly(lactic-co-glycolic acid)	PLGA
Mesoporous silica nanoparticles	MSNs
Amphiphilic poly(lactic-co-glycolic acid)–polyethylene glycol	PLGA-PEG
Superparamagnetic iron oxide nanoparticles	SPIONs
Adipic acid dihydrazide	ADH
Hyaluronic acid	HA
Erlotinib	ERL
Bevacizumab	BEV
Transferrin	Tf
Methoxy poly (ethylene glycol)-poly(ethylenimine)-poly(l-glutamate)	mPEG-OEI-PLG
Doxorubicin	DXR
Cisplatin	CP
Tocopherol polyethylene glycol 1000 succinate	TPGS
Polyvinyl imine	PEI
Polycaprolactone	PCL
Polyvinyl alcohol	PVA
Poly (β-Amino ester)	PBAE
Near-infrared	NIR
Conjugated linoleic acid	CLA
Lipid-coated iron oxide NPS	IONP-C/O@LP
Cytosine-phosphate-guanine	CPG
Genistein	GNS
All-trans retinoic acid	ATRA
PGAM1 siRNA	siPGAM1
Multidrug resistance	MDR
P-glycoprotein	P-gp
General control non-repressed 5 protein	GCN5
Inhibitors of apoptosis proteins	IAPs
Small hairpin RNAs	shRNA
Hypoxia-inducible factors	HIFs
ATP-binding cassette	ABC
Caspase 8	CASP8
Liposome-incorporated poly	ε-caprolactone
